# Virome profiling of *Culex tarsalis* through small RNA-seq: A challenge of suboptimal samples

**DOI:** 10.1371/journal.pntd.0013611

**Published:** 2025-11-03

**Authors:** Jaime Manzano-Alvarez, Sultan Asad, Duverney Chaverra-Rodriguez, Eunho Suh, Jason L. Rasgon

**Affiliations:** 1 Department of Entomology, The Pennsylvania State University, University Park, Pennsylvania, United States of America; 2 Center for Infectious Disease Dynamics, The Pennsylvania State University, University Park, Pennsylvania, United States of America; 3 Dirección Académica, Universidad Nacional de Colombia Sede de La Paz, La Paz, Colombia; 4 Department of Biochemistry and Molecular Biology, The Pennsylvania State University, University Park, Pennsylvania, United States of America; 5 The Huck Institutes of the Life Sciences, The Pennsylvania State University, University Park, Pennsylvania, United States of America; 6 The ONE Health Microbiome Center, The Pennsylvania State University, University Park, Pennsylvania, United States of America; 7 Institute of Energy and the Environment, The Pennsylvania State University, University Park, Pennsylvania, United States of America; Connecticut Agricultural Experiment Station, UNITED STATES OF AMERICA

## Abstract

Viral infections in mosquitoes trigger the RNA interference (RNAi) pathway, a key antiviral defense mechanism that generates virus-derived small RNAs (vsRNAs). Given the natural enrichment of vsRNAs during infection and their stability, small RNA sequencing (sRNA-seq) has emerged as a powerful tool for virome characterization. *Culex tarsalis* is a widely distributed mosquito species in North America and is an important vector of West Nile virus (WNV). Previous studies have shown that co-infection with insect-specific viruses (ISVs) can modulate WNV replication in *Cx. tarsalis*, highlighting the importance of characterizing the virome of this species. Here, we investigated the virome of *Cx. tarsalis* populations across 5 states of the Western United States using sRNA-seq. We analyzed samples from 17 geographic locations which were collected under suboptimal field conditions during the COVID-19 pandemic, presenting challenges related to sample integrity. Despite these challenges, sRNA-seq proved to be a reliable method for virome analysis. We identified a total of seven ISVs, all of which have been previously associated with *Cx. tarsalis*, along with their respective sRNA (siRNA and piRNA) profiles. The ISVs found here did not show a clear distribution pattern, but two of them (Marma virus and *Culex* narnavirus 1) were found in all sampled states. These findings not only deepen our understanding of ISVs, but also demonstrate the utility of sRNA-seq in non-ideal situations, enabling the collection and analysis of samples under real-world surveillance scenarios.

## Author summary

Mosquitoes are capable of transmitting pathogenic viruses to humans and other vertebrate hosts during feeding. However, they can also carry viruses that infect only mosquitoes, which are known as insect specific viruses (ISVs). Some ISVs are have the potential to affect how mosquitoes interact with viruses that can cause diseases in vertebrates. When a virus infects a mosquito, the insect’s immune system responds by producing small RNA fragments complementary to the virus’s genetic code. In this study, we sequenced these fragments and assembled them to identify seven ISVs present in *Culex tarsalis* mosquitoes collected under non-ideal storage conditions from five USA states. It is important to note that although the samples were not collected under ideal conditions for RNA studies, valuable information was still recovered by studying the small RNAs. Our work highlights the diversity of ISVs that *Cx. tarsalis* mosquitoes carry, and demonstrates how even suboptimal samples can provide insights into mosquito viral communities and their relationships.

## Introduction

Insect specific viruses (ISVs) are viruses that infect insects, but lack the ability to replicate in vertebrate cells [1,2]. Due to host restriction, high prevalence, and potential to modulate arbovirus infections, they have gained attention as potential biocontrol agents and as tools for studying vector biology, as well as potential agents for disease control [3–11]. In the case of mosquitoes, their virome includes ISVs and pathogenic arboviruses, the latter being able to infect vertebrates. Mosquito ISVs can interact with pathogenic arboviruses such as West Nile virus (WNV; *Flaviviridae*) [12,13]. WNV is considered the leading cause of arboviral disease in the United States, with more than 1200 neuroinvasive cases and around 120 deaths reported each year [14]. Eilat virus (EILV: *Togaviridae*), an ISV naturally found in *Culex univittatus*, *Cx. pipiens* and *Anopheles coustani* [15–17], can induce superinfection exclusion against WNV in *Cx. tarsalis* mosquitoes in a strain specific manner by reducing the WNV viral titers in the bodies of mosquitoes previously infected with EILV [5]. Thus, their potential role in affecting the replication dynamics of human arboviruses highlights the public health significance of studying ISVs in this mosquito species. More than 39 ISVs have been discovered in *Cx. tarsalis* mosquitoes or cell cultures, but most virome studies for this species are restricted to California and Iowa [18–20]. Consequently, even though this species is distributed across North America, its virome remains unexplored across the majority of its range.

sRNAs are short, non-coding RNA molecules that can play critical roles in gene regulation and antiviral defense mechanisms [21]. In mosquitoes, sRNAs are key players in the RNA interference (RNAi) pathway, an important component of the innate immune system that targets and degrades viral RNA. Some classes of sRNAs that participate in RNAi viral response, include small interfering RNAs (siRNAs; 21nt), and piwi-interacting RNAs (piRNAs; 24–30nt), which are involved in different biogenesis pathways and have different biological functions [22–25]. The sRNAs produced during RNAi response against viral infection are considered virus-derived small RNAs, and are the focus of virome studies because they provide a molecular signature of viral presence. Thus, the application of sRNA-seq in mosquito virome studies allows for the identification of known and novel viruses [26–29], as well as a unique virus molecular signatures, or sRNA profiles, which could be used for identification and understanding of their interactions within their host (reviewed in [30]). Importantly, since small RNAs (sRNAs) are naturally enriched during viral infections in insect cells, the use of sRNA-seq has proven to be an efficient, specific and reliable approach in previous studies [26–28,30–32].

While both RNA-seq and sRNA-seq have been used for virome identification in mosquitoes [4,18,26,30,31], sRNA-seq has advantages due to the natural enrichment of sRNAs during viral infections, as well as their higher stability due to their small size, association with proteins, and 2′-O-methylation at the 3′ terminal which protects them from degradation by exonucleases [30,33–35]. COVID-19 pandemic mitigation strategies caused a wide range of challenges for researchers, impacting both field and lab work [36]. To address this challenge, we collaborated with mosquito control agencies that routinely collect mosquitoes for surveillance purposes across multiple locations in the United States. Since trapping and shipping methods do not always allow for strict adherence to cold-chain protocols, preserving RNA integrity can be difficult. Thus, we took advantage of the stability of sRNA and used a sRNA-seq approach. In this study, we investigated the virome of *Cx. tarsalis* mosquitoes collected from 17 geographic locations across five Western states using sRNA sequencing and bioinformatic analyses.

## Methods

### Sample collection

Wild *Cx. tarsalis* mosquitoes were collected from 17 different locations across 5 states in the USA: California (CA), Colorado (CO), Texas (TX), Washington (WA), Utah (UT). Three locations per state were sampled, except for CA and TX with 5 and 2 locations respectively. Mosquitoes were trapped using CDC (Centers for Disease Control miniature light trap) and BG (Biogents Sentinel trap) traps, then identified and immediately stored in DNA/RNA shield (Zymogen Cat. No. R1200) by Vector Disease Control International (VDCI), and local mosquito control agents. Mosquitoes were shipped at room temperature to the Rasgon Lab at The Pennsylvania State University, where they were sexed and immediately frozen at -80°C until further processing. Additionally, one pool of three virus-free female colony *Cx. tarsalis* mosquitoes (KNWR strain) from the Rasgon lab was used as a negative control for virus detection.

### RNA extraction

RNA was extracted from pools of 3 female *Cx. tarsalis* mosquitoes per geographic location (male mosquitoes were not considered for this study) using Qiazol lysis Reagent (Qiagen Cat. No./ ID: 79306) and Direct-zol RNA MicroPrep kit (Zymogen Cat. No. R2062). Briefly, mosquitoes were thawed in DNA/RNA shield solution and were rinsed once in 1x phosphate buffered saline (PBS). 1000ul of Qiazol and 0.2-0.5 nm metallic beads were added to the pools in 1.5ml tubes, then samples were homogenized using Qiagen tissue lyser for 90 second at 30.0 hertz/second, and then centrifuged at 15000 xg for 1 minute. The clear lysate was transferred to a new 2ml tube and RNA was purified using Direct-zol RNA MicroPrep kit using manufacturer instructions. Extracted RNA was sent to Novogene USA to perform small RNA sequencing.

### Library preparation and sequencing

For each sample, RNA quantity was measured using Qubit RNA HS assay (ThermoFisher), and RNA quality was assessed using Bioanalyzer 2100 Eukaryote total RNA Nano (Agilent Technologies, CA, USA). Total RNA was used as input material for RNA sample preparations, and library preparation was performed with the NEB Next Small RNA Library Prep Set for Illumina (18–40 bp insert size). Briefly, 3′ and 5′ adaptors were ligated to the 3′ and 5′ ends of small RNA, respectively. Then, the first-strand cDNA was synthesized after hybridization with the reverse transcription primer. The double-stranded cDNA library was generated through PCR enrichment.

After purification and size selection, libraries with insertions between 18–40 bp were ready for sequencing. Upon completion of library construction, the concentration of the inserted fragments was quantified using Qubit 2.0 DNA HS Assay (ThermoFisher) with QuantStudio 5 System (Applied Biosystems, USA), and Tapestation high sensitivity D1000 assay (Agilent Technologies, CA, USA) to ensure library quality. The qualified libraries were pooled and sequenced on the Illumina platform to generate ~20 million single-end 50 bp reads per sample. The original fluorescence image files obtained from the Illumina platform were transformed into short reads (Raw data) by base calling. These short reads are recorded in FASTQ format [37], which contains sequence information and corresponding sequencing quality information.

### Small RNA reads preparation

Raw single end sequences were trimmed using cutadapt (v5.0), removing reads with average Phred quality below 20, ambiguous nucleotides, and reads with length shorter than 15nt and/or without adaptors. The remaining reads were mapped to the *Cx. tarsalis* reference genome CtarK1 [38] using bowtie2 (version 2.5.1), and bacterial reference genomes via Kraken2 (version 2.1.3). Reads that did not map to mosquito or bacteria reference genomes were then considered as processed reads and were used for further steps, since they could be of viral origin.

### Contig assembly and extension

Processed reads that were 20–30nt long were used for contig assembly in each library (pool per location) using Velvet (version 1.2.10) and Spades (version 3.13.0), as previously described [31]. Contigs >200nt from each location were grouped using CD-HIT (version 4.8.1) with at least 90% coverage and 90% identity in order to remove redundancy, and then compared against the NCBI NT and NR databases with BLAST. Contigs that were identified as viral or unknown origin were chosen, and then sRNA profiles and coverage of each contig were plotted using in-house Perl v5.16.3, BioPerl library v1.6.924 and R v4.3.2 scripts, as previously described [26]. Contigs were manually curated by selecting those that showed a 21nt peak in their sRNA profiles, indicative of Dicer-processed sRNA species. Contigs with mapped reads displaying even coverage in both forward and reverse orientations were selected. Filtered contigs from every location were properly labeled and grouped together with CD-HIT to designate representative contigs. Contigs were then mapped against the processed reads for co-occurrence analyses based on hierarchical clustering with Pearson correlation using R scripts. Contigs that clustered together were used for contig extension in Spades as trusted contigs with all the libraries that mapped against them (minimum of 300 reads). All the extended contigs were grouped together and then manually curated. Contigs that had their top three BLAST matches against viral sequences of the same species were used to identify their likely origin. Finally, reference genomes of the identified viruses were subsequently used as templates for sRNA profiling, genome coverage and co-occurrence analyses as previously described.

### RT-PCR for virus detection

Pools of three female *Cx. tarsalis* mosquitoes per location were prepared. Samples were collected in 2022 from different locations of California and Colorado following the methods described above. Additionally, one pool of three female *C. tarsalis* KNWR mosquitoes from the laboratory insectary was included as a negative control. Total RNA was extracted using the RNeasy Mini Kit (Qiagen), and 1 µg of RNA was treated with DNase I RNase-free (ThermoFisher Scientific) to remove residual genomic DNA. Complementary DNA (cDNA) was synthesized from 1 µg of DNA-free RNA using the qScript cDNA Synthesis Kit (Quantabio). Target viral RNA sequences were amplified with the Phusion High-Fidelity DNA Polymerase PCR Kit (New England Biolabs). Amplicons were visualized on a 2% agarose gel, and specific bands were excised and purified with the Monarch Spin DNA Gel Extraction Kit (New England Biolabs). Purified PCR products were submitted for Sanger sequencing, and BLAST was used to match the sequences to their likely origin.

### Data reporting

RNA libraries are deposited at https://zenodo.org/records/15412939

### Use of AI

ChatGPT and GitHub Copilot were used to assist in generation of some of the pipeline code used for data analysis. Generated code was tested, debugged, and validated manually. All codes and scripts are available at https://github.com/Zureishon/Virome.

### Figure generation

Graphs and plots were made with R Studio (2023.3.0.386; PBC, Boston, MA, USA) and Biorender.com. Final figures were assembled using Adobe Illustrator 2023 (27.4.1; Adobe, San Jose, CA, USA). Statistical summaries of RNA libraries and assembled contigs are provided as supplemental material.

## Results

### Seven ISVs Identified in Culex tarsalis via sRNA-Seq

**Fig 1:**
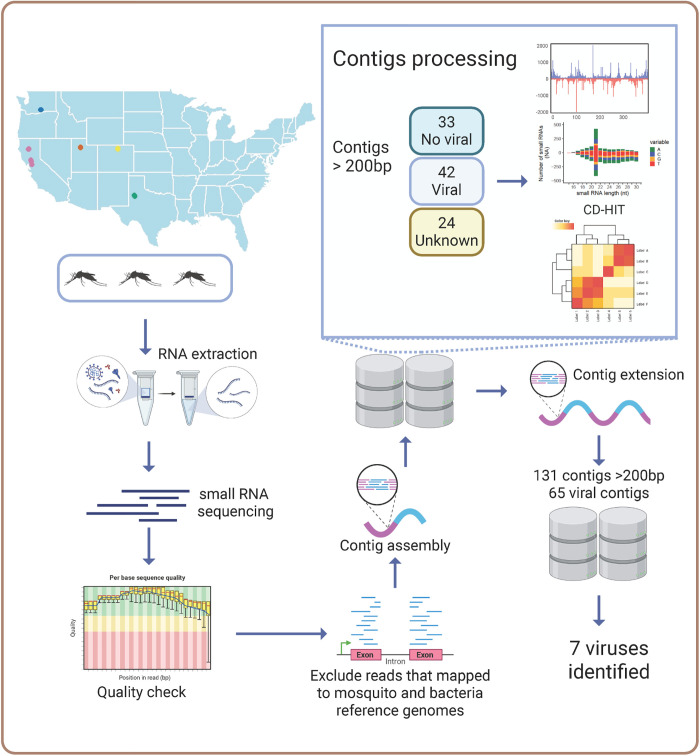
Graphical methods of metagenomics pipeline for virome profiling. Pools of 3 female mosquitoes per location were used for RNA extraction and sRNA-seq. Raw reads underwent quality checks and were aligned to mosquito and bacterial genomes, then unmapped reads were used for contig assembly. Viral and unknown contigs were used for co-occurrence analyses and contig extension. Extended contigs were analyzed by BLAST for virus identification, and available viral reference sequences were used for virus co-occurrence analyses and sRNA profile design. Figure created in BioRender. Rasgon, **J.** (2025) https://BioRender.com/o2bfaiw and with RStudio (map figure; libraries ggplot2 and maps).

**Fig 2:**
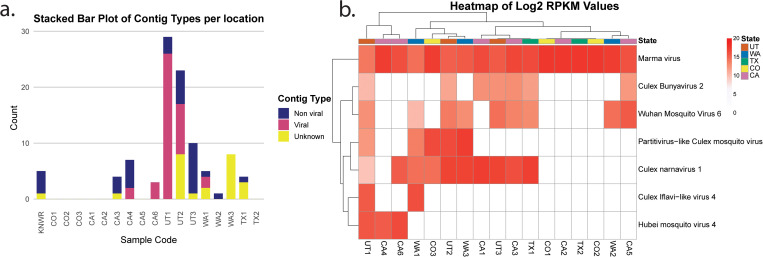
(a) Contig classification per sample and ISV distribution. For each library, assembled contigs longer than 200 bp were analyzed by BLAST and classified according to the closest BLAST hit. (b) Heatmap shows the Log₂ RPKM values for each library, based on mapping to reference genomes of ISVs identified through metagenomics analyses.

**Fig 3:**
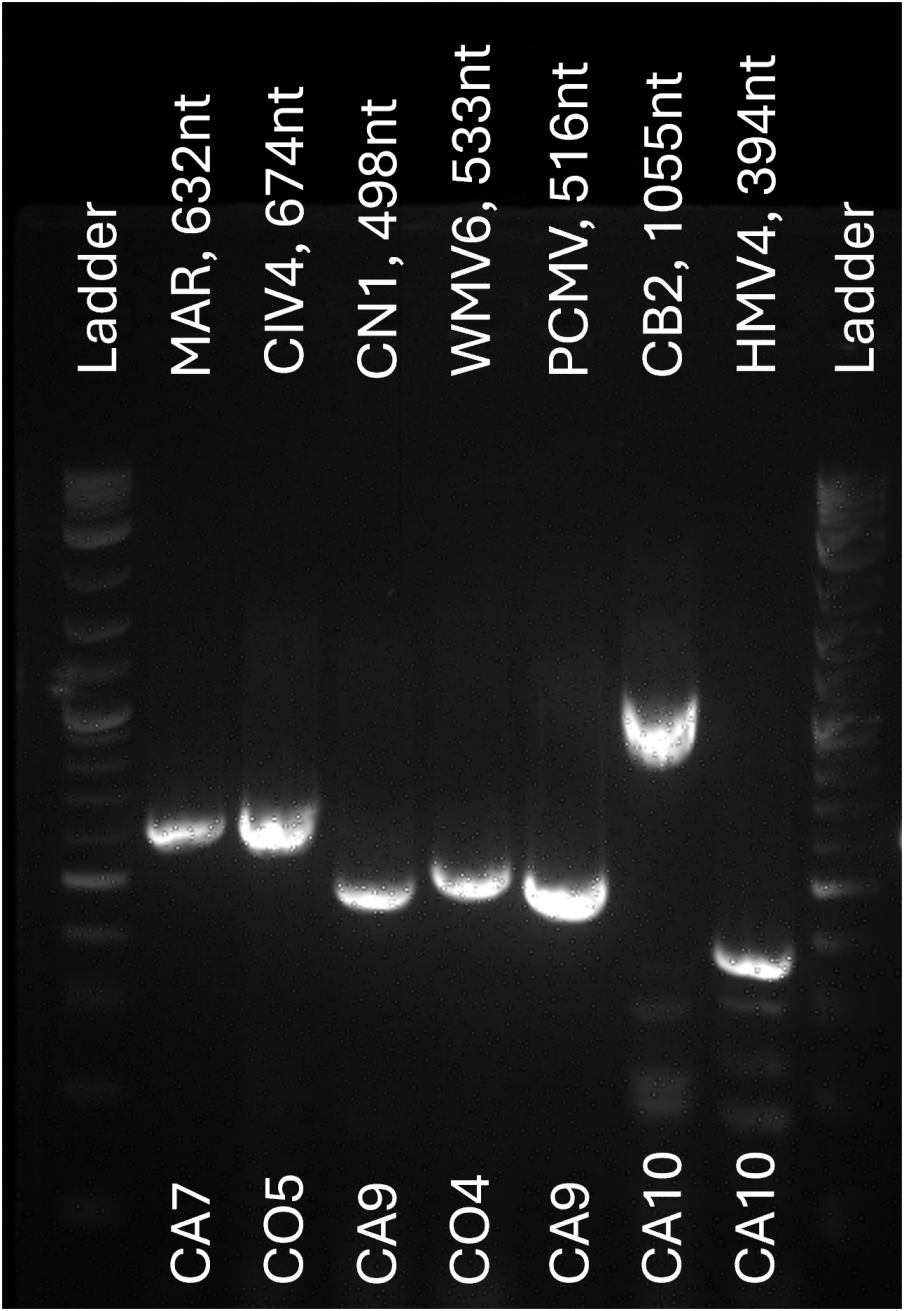
Agarose gel electrophoresis of representative RT-PCR products. PCR products from each representative sample were gel-extracted and purified for Sanger sequencing. Primer names and expected amplicon sizes are indicated above the lanes, and sample locations of origin are shown below the lanes. A DNA ladder is included for size reference. The full set of RT-PCR screening gels, including positive controls, is provided in S3 Fig. Details for each primer set are provided in Table 2.

From each of the 17 locations, one pool of three unfed female *Cx. tarsalis* mosquitoes was processed for RNA extraction and sRNA-seq (Fig 1). Due to suboptimal collection conditions for RNA preservation, challenges in RNA extraction and integrity were expected. This is shown in the variation of total RNA, and RNA integrity numbers (RIN) across samples (S1 Table). Moreover, post-trimming, library sizes varied between 3–25 million (M) reads, reflecting differences in sRNA availability across samples. Most libraries contained fewer than 2M reads within the target sRNA size range (20–29 nt), with the exception of UT1, UT2, and UT3, which exceeded 4M target reads (S1a Fig). Reads were first mapped to the *Cx. tarsalis* genome and bacterial sequences for filtering. In most libraries, over 80% of reads mapped to the *Cx. tarsalis* genome; however, in CO2, WA1, WA3, and TX2, less than 45% mapped, suggesting higher proportions of non-host sequences (S2b Fig). Given the limited size of the libraries, we employed two assembly strategies using Velvet and SPAdes to maximize the contig assembly. We identified 99 contigs longer than 200 bp, which were subsequently analyzed by BLAST to determine their origins. Contigs of viral origin were detected in 5 out of the 17 sampled populations (Fig 2a). To refine our virome analysis, we examined sRNA profiles and coverage maps of contigs classified as viral or of unknown origin. Contigs displaying a 21-nt peak and even coverage were used for further analysis (S2a Fig). From this selection, 26 representative contigs were chosen for co-occurrence analyses based on their RPKM (reads per kilobase million) values, which grouped samples into 8 distinct clusters (S2b Fig). These clusters, along with their associated representative contigs, were then subjected to a second round of contig assembly using SPAdes. This approach yielded 131 unique contigs longer than 200 bp with a maximum of 1387 bp, of which 65 contigs were identified as viral in origin, corresponding to seven ISVs. To determine the closest viral references, all contigs were analyzed by BLAST, and available reference genomes were used for co-occurrence analyses (Fig 2b). The most widely distributed viruses were Marma virus (MV) and *Culex* narnavirus 1 (CN1), present across all five states. Wuhan Mosquito virus 6 (WMV6) detected in four states, while *Culex* Bunyavirus 2 (CB2) and Partitivirus-like *Culex* mosquito virus (PCMV) were found in three states. Lastly, *Culex* Iflavi-like virus 4 (CIV4) and Hubei mosquito virus 4 (HMV4) were identified in two states (Fig 2b). All detected viruses were classified as RNA viruses (Table 1), all of which have been previously reported in *Cx. tarsalis* mosquitoes from other areas [39]. No viral reads were detected in the KNWR insectary sample thus it was excluded from subsequent analyses. Virus presence in field-collected mosquitoes was further corroborated by RT-PCR (Figs 3 and S3) and Sanger sequencing (S2 Table) in samples from 2022. Primer sequences used for amplification are provided in Table 2.

**Table 1:** Classification and previous reports of characterized viruses in North America. GenBank accession numbers refer to the viral reference sequences used for the assembly of sRNA profiles. The states where each virus was detected are indicated.

Genome type	Viral family	Virus name	Number of samples detected	Reference sequence used (Accession number)	States	Previously reported in North America
Single stranded positive-sense RNA	*Iflaviridae*	*Culex iflavi-like virus 4*	4	MH188011.1	UA, WA	[18,39,42]
	*Narnaviridae*	*Culex narnavirus 1*	10	MK628543.1	UT, WA, TX, CO, CA	[39,42]
	*Tombusviridae*	*Hubei mosquito virus 4*	3	KX883008.1	UT, CA	[39,42]
		*Marma virus*	17	MW434901.1*	UT, WA, TX, CO, CA	[39,42]
Single stranded negative-sense RNA	*Orthomyxoviridae*	*Wuhan mosquito virus 6*	9	MF176248.1†	UT, WA, TX, CA	[39,42]
	*Peribunyaviridae*	*Culex bunyavirus 2*	7	MH188052.1	UT, TX, CA	[18,39,42]
Double stranded RNA	*Partitiviridae*	*Partitivirus-like Culex mosquito virus*	5	MH188050.1	UT, WA, CO	[18,39,42]

*The closest and longest BLAST hit sequence was used when reference genome sequences were not available. † MF176248.1 to MF176253.1 were used (all of them are part of the GenBank assembly GCA_031525425.1).

**Table 2:** List of primers used for viral detection. Primers were designed with primer-BLAST and SnapGene.

Virus	Accession number	Primer	Sequence (5′ → 3′)	Target region
Culex iflavi-like virus 4	MH188011.1	CIV4-F	CGTGTTTCAGGATTGTTTG	8550-8568
		CIV4-R	TTCCATCATCACCATACACG	9204-9223
Culex narnavirus 1	MK628543.1	CN1-F	CACGACAATCTGGCTCATAG	1417-1436
		CN1-R	GAAGCTTTTCAGGCGATTTG	1895-1914
Hubei mosquito virus 4	KX883008.1	HMV4-F	GAACCAAAAGGCGATGACCG	124-143
		HMV4-R	TCGATACAGTCCGGTTCCCT	498-517
Marma virus	MW434901.1	MAR-F	CAAGAGCCGCATAAACTGAA	2207-2226
		MAR-R	CAATTTTGGACTTTAGCCCC	2819-2838
Wuhan mosquito virus 6	MF176249.1	WMV6-F	GGTTCCAAGATGATGTGCTT	1614-1633
		WMV6-R	TTCCTCAGTCTTGTCTTGGT	2127-2146
Culex bunyavirus 2	MH188052.1	CB2-F*	GAGTCCTTGTCCATCCCCGC	991-1010
		CB2-R*	GTGCAGGAAGAAGTAGCATGG	2025-2045
Partitivirus-like Culex mosquito virus	MH188050.1	PCMV-F	GGATTCGCCATACGAAGAAA	524-543
		PCMV-R	ACTCGGTCAAATCGATACCT	1020-1039
Ctar-792 40S ribosomal protein (Control)	EZ000607.1	CON-F	CTGGAGATGAACTCGGACCT	1-20
		CON-R	TTACAGGTAAGGTTCCGGGAAC	474-495

*Primers are a modified version of “Bunyaviridae environmental sample” from [63]

**Fig 4:**
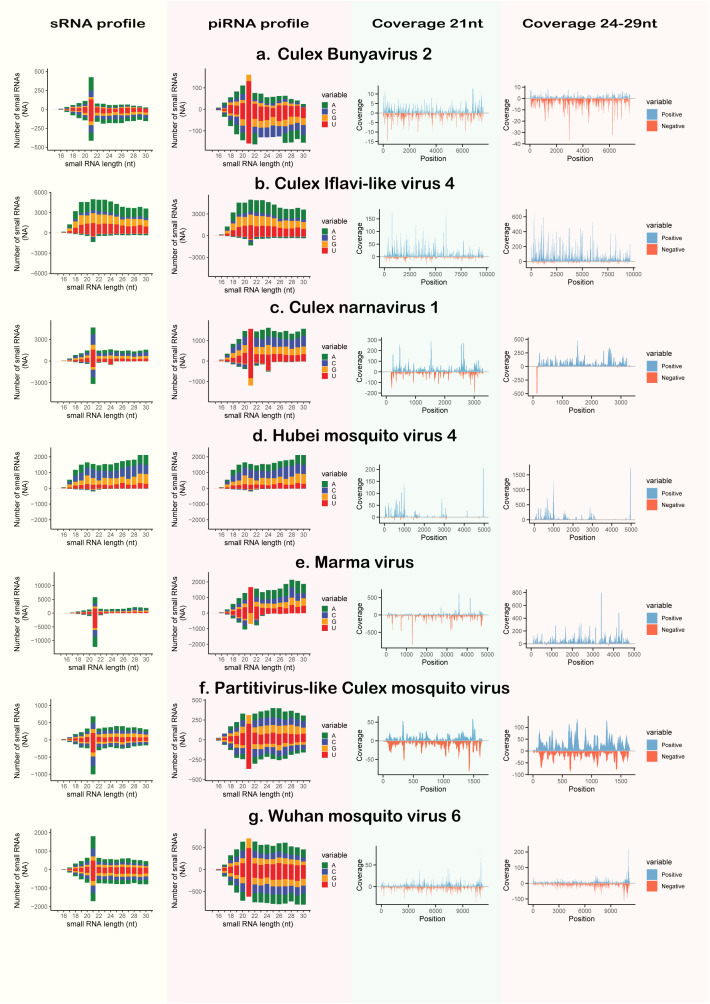
sRNA profiles and coverage plots of the ISVs. All libraries were used to generate these graphs. sRNA profiles are histograms representing the frequency of mapped read sizes, with colors indicating 5’ nucleotide bias. piRNA profiles are sRNA profiles filtered to include only reads with a 5’ U bias or an A at the 10th nucleotide. Coverage plots were generated using reads of either 21 nt in length, representing siRNAs, or reads of 24–29 nt, representing piRNAs. Colors in the coverage plots indicate whether the reads aligned to the sense (blue) or antisense (red) strand of the reference viral genomes.

We then analyzed sRNA profiles and coverage maps for each identified virus. sRNA profiles are histograms displaying the absolute number of reads per size that align to the genome in the forward and reverse orientations; piRNA profiles follow the same principle but only include reads enriched for U at the 5’ end or A at the 10th nucleotide. Coverage maps illustrate the depth of alignment by showing how many times each nucleotide in the genome is covered by a read, plotted separately for 21-nt reads and 24–29-nt reads. Most of the identified viruses showed a peak in the 21-nt size reads, indicative of siRNA pathway activation, with participation of the piRNA pathway represented by the presence of 24–30nt reads. However, CIL4 and HMV4 exhibited an enrichment of sRNA reads mostly in the forward sense, with almost identical sRNA and piRNA profiles. Coverage maps revealed that sRNAs were mapped across most of each viral genome, except for HMV, which displayed regions with little to no read coverage (Fig 4).

## Discussion

RNA-seq has become a powerful tool for the surveillance and discovery of new viruses in insects. In mosquitoes, its use has been demonstrated across several countries, especially in species of the genus *Aedes* [26,28,30–32,40,41]. In North America, some studies have analyzed the virome of *Culex* mosquitoes using either long or small RNA-seq, with most focusing on Canada, and the states of California and Iowa in the USA. The majority of these studies have examined *Cx. pipiens* or *Cx. quinquefasciatus*, with only three investigations including *Cx. tarsalis* [18,19,39,42,43]. In this study, we detected 7 of the 39 viruses associated with *Cx. tarsalis* from California [18] using sRNA-seq, and corroborated these results through RT-PCR on samples from a subsequent year, confirming the accuracy of this sRNA-seq-based viral identification. These viruses are presumed to be ISVs, detected only in mosquitoes and other insects, with no confirmed vertebrate infections. Importantly, their ability to replicate in vertebrate cells has not been tested. In contrast, the majority of mosquito-borne arboviruses are members of the families *Flaviviridae* (order Amarillovirales), *Togaviridae* (order Martellivirales), and *Peribunyaviridae* or *Phenuiviridae* (order Bunyavirales) [44–47]. Although CB2 has been associated with *Peribunyaviridae* [18], there is no evidence to date that it infects vertebrate hosts, and it is therefore currently considered an ISV. Moreover, the impact of these viruses on coinfection dynamics with arboviruses remains to be experimentally explored.

The use of sRNA-seq enables the exploration of the sRNA profiles, which can reflect how viruses interact with the mosquito immune response. In most cases, the sRNA profiles in this study resemble previously described profiles for each virus [42], except for HMV4, and PCMV. These results support the reliability of sRNA profiles, even under potentially different environmental and physiological conditions. Importantly, we observed the activation of the siRNA pathway in response to most viruses, which is expected since the siRNA pathway is regarded as the main antiviral response in mosquitoes [48]. It is typically triggered by viral double-stranded RNA, which is a common byproduct of viral infections [49], cleaving it into 21-nt siRNAs that guide the degradation of viral genomes [50,51]. In contrast, HMV4 and CIV4 elicited a stronger piRNA pathway response compared to siRNA.

The piRNAs primarily originate from the mosquito’s own genome, producing slightly longer sRNAs (24–30 nt) from piRNA clusters [22,52,53]. These piRNA clusters are likely derived from endogenous viral elements formed during previous viral infections, and this pathway has been regarded as a mechanism of long-lasting and transgenerational mosquito adaptive immunity [54]. Moreover, this pathway participates in the antiviral response during acute and persistent viral infections in mosquitoes [55]. In most cases, the piRNA clusters are transcribed from a single DNA strand, leading to a characteristic strand bias in piRNAs [56,57]. Activation of those pathways have been inferred for several viruses infecting *Culex* mosquitoes, both *in vivo* and *in vitro* [20,50,58,59], as well as in virome-wide analyses via sRNA-seq [42]. Coverage plots indicate that most sRNAs mapping to HMV4 and CIV4 were strand-biased, aligning predominantly to a single strand, consistent with a piRNA response and impairment of siRNA response (e.g., Dicer inhibition) [30,50,60].

An important aspect of this study is that mosquitoes were collected by mosquito control agencies as part of their routine surveillance, using overnight traps, during an active pandemic that limited ability to ship or process samples in a timely manner. Under these conditions, it is difficult to guarantee high-quality RNA, as mosquitoes may die before collection or processing is complete, leading to RNA degradation. In this study, most samples had a RIN below 7, which is a common threshold suggested by sequencing facilities. Although there is not a strict threshold for RIN, lower values have been associated with RNA decay, which could affect upstream analyses [61,62]. To overcome the RNA degradation in our samples, we took advantage of the higher stability of small sRNAs. The sRNA libraries generated in this study were, in most cases, around five times smaller than those used in previous work [26,32,41]. As a result, the assembled viral contigs were relatively short, limiting our ability to reconstruct full viral genomes. Nonetheless, we were able to accurately identify viral infections, highlighting the potential of sRNA-based approaches for RNA virus surveillance in mosquitoes (or other insects) even when collection conditions are suboptimal. Improvements for further studies could include better coordination with mosquito control agencies to enhance sample preservation, as well as the adoption of more efficient RNA extraction methods. These adjustments could significantly improve RNA quality and increase the likelihood of assembling complete viral genomes, capture more viral diversity, or the identification of novel viruses.

In conclusion, this study demonstrates the utility of sRNA-seq for virus surveillance in *Cx. tarsalis,* providing new insights into the mosquito anti-viral immune response. Despite challenges related to sample integrity, we observed activation of both siRNA and piRNA pathways in response to most viruses, with HMV and CIV4 triggering only a piRNA response. Moving forward, expanding sample collection efforts and refining preservation and extraction methods will strengthen future virome surveillance studies.

## Supporting information

S1 Data Database containing raw data summaries, and Sanger sequencing results. (XLSX)

S2 Data Contigs FASTA dataset and associated BLAST outputs. (ZIP)

S1 Fig Library size distribution and read classification per location. (a) Reads from each library were classified by size, with 20–30 bp representing the desired range for sRNA sequencing. (b) Relative numbers of reads mapped to mosquito, identified viruses, and unknown reads that did not map to either category.
(DOCX)

S2 Fig Contig processing, heatmap and clustering of representative contigs. (a) Flow chart the inputs, outputs, and programs used for contig processing. (b) Libraries were mapped against the representative contigs and the number of reads mapped per contig was used to calculate the Log2 RPKM values. Library clusters, and their corresponding contigs, were used for contig extension.
(DOCX)

S3 Fig Screening agarose gel electrophoresis of RT-PCR products. Primers used are listed in Table 2. Pop 1–6 correspond to locations CA7, CA8, CA9, CA10, CO4 and CO5, respectively, while C corresponds to a sample of Cx. tarsalis KNWR strain from the laboratory insectary. Primers were used to assess viral presence in different samples, with control gene primers included as a positive control (in b. and c. the control gene primers were used in the mosquito laboratory sample, or “C” pool). Once viral presence was confirmed, a final RT-PCR was performed to gel-extract the products for Sanger sequencing, as shown in Fig 3 and S2 Table.
(DOCX)

S1 Table Pre-sequencing RNA quality control results provided by the sequencing facility (Novogene). The RNA integrity number (RIN) is reported as an indicator of RNA quality, with higher values reflecting more intact RNA. RNA quality was assessed with Bioanalyzer 2100 Eukaryote total RNA Nano.
(DOCX)

S2 Table BLAST analysis of Sanger sequencing results using blastn against the NCBI core nucleotide database. The table reports the closest hits with their accession numbers.
(DOCX)
