# Supplementary material for: Virome profiling of Culex tarsalis through small RNA-seq: A challenge of suboptimal samples
Source: PLoS Negl Trop Dis. 2025 Nov 3;19(11):e0013611. doi: 10.1371/journal.pntd.0013611 (PMC12591400; doi:10.1371/journal.pntd.0013611)
Supplement: S1 Fig — (a) Reads from each library were classified by size, with 20–30 bp representing the desired range for sRNA sequencing. (b) Relative numbers of reads mapped to mosquito, identified viruses, and unknown reads that did not map to either category. (DOCX) [file pntd.0013611.s003.docx]

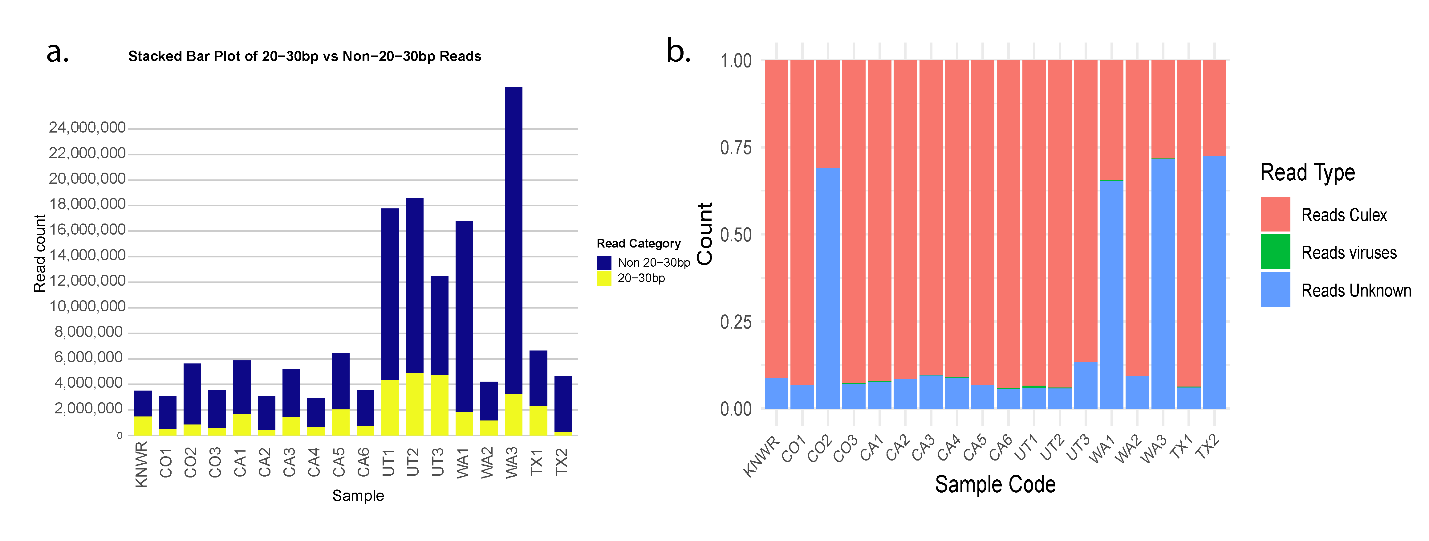


S1 Fig. Library size distribution and read classification per location. (a) Reads from each library were classified by size, with 20–30 bp representing the desired range for sRNA sequencing. (b) Relative numbers of reads mapped to mosquito, identified viruses, and unknown reads that did not map to either category.
