# Supplementary material for: Virome profiling of Culex tarsalis through small RNA-seq: A challenge of suboptimal samples
Source: PLoS Negl Trop Dis. 2025 Nov 3;19(11):e0013611. doi: 10.1371/journal.pntd.0013611 (PMC12591400; doi:10.1371/journal.pntd.0013611)
Supplement: S2 Fig — (a) Flow chart the inputs, outputs, and programs used for contig processing. (b) Libraries were mapped against the representative contigs and the number of reads mapped per contig was used to calculate the Log2 RPKM values. Library clusters, and their corresponding contigs, were used for contig extension. (DOCX) [file pntd.0013611.s004.docx]

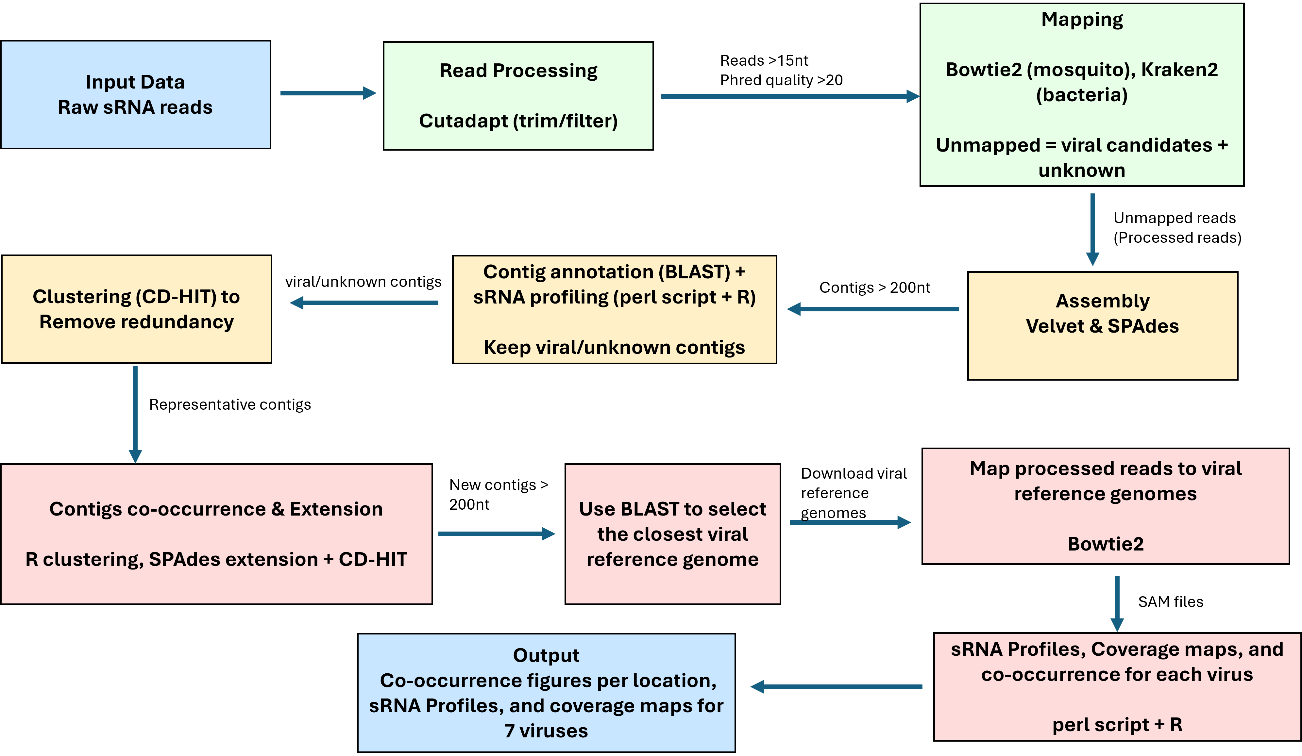


**a.**


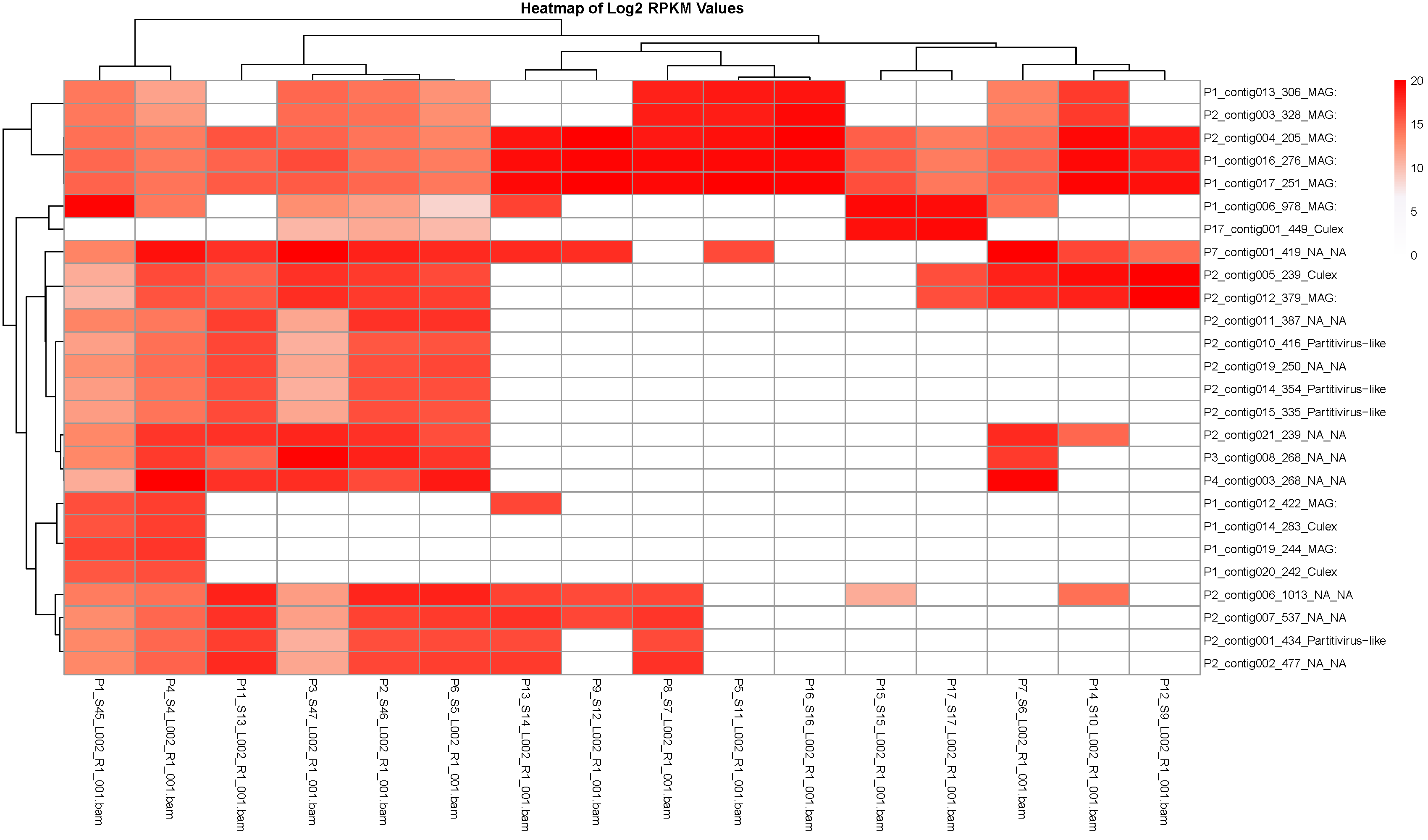


**b.**

S2 Fig. Contig processing, heatmap and clustering of representative contigs. (a) Flow chart the inputs, outputs, and programs used for contig processing. (b) Libraries were mapped against the representative contigs and the number of reads mapped per contig was used to calculate the Log2 RPKM values. Library clusters, and their corresponding contigs, were used for contig extension.
