# Supplementary material for: Virome profiling of Culex tarsalis through small RNA-seq: A challenge of suboptimal samples
Source: PLoS Negl Trop Dis. 2025 Nov 3;19(11):e0013611. doi: 10.1371/journal.pntd.0013611 (PMC12591400; doi:10.1371/journal.pntd.0013611)
Supplement: S3 Fig — Primers used are listed in Table 2. Pop 1–6 correspond to locations CA7, CA8, CA9, CA10, CO4 and CO5, respectively, while C corresponds to a sample of Cx. tarsalis KNWR strain from the laboratory insectary. Primers were used to assess viral presence in different samples, with control gene primers included as a positive control (in b. and c. the control gene primers were used in the mosquito laboratory sample, or “C” pool). Once viral presence was confirmed, a final RT-PCR was performed to gel-extract the products for Sanger sequencing, as shown in Fig 3 and S2 Table. (DOCX) [file pntd.0013611.s005.docx]

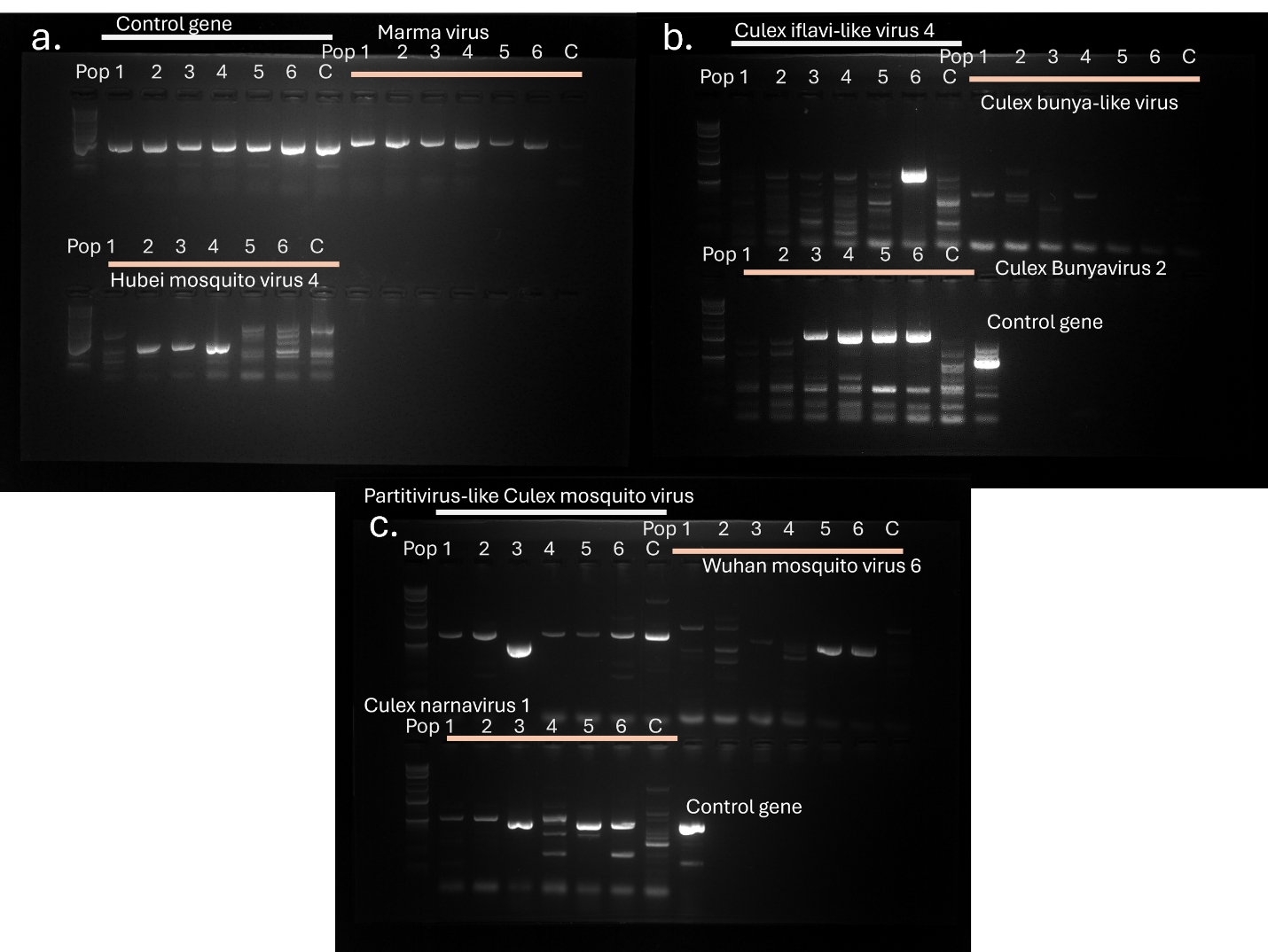


S3 Fig. Screening agarose gel electrophoresis of RT-PCR products. Primers used are listed in Table 2. Pop 1–6 correspond to locations CA7, CA8, CA9, CA10, CO4 and CO5, respectively, while C corresponds to a sample of *Cx. tarsalis* KNWR strain from the laboratory insectary. Primers were used to assess viral presence in different samples, with control gene primers included as a positive control (in b. and c. the control gene primers were used in the mosquito laboratory sample, or “C” pool). Once viral presence was confirmed, a final RT-PCR was performed to gel-extract the products for Sanger sequencing, as shown in Fig 3 and S2 Table.
