# Supplementary material for: Virome profiling of Culex tarsalis through small RNA-seq: A challenge of suboptimal samples
Source: PLoS Negl Trop Dis. 2025 Nov 3;19(11):e0013611. doi: 10.1371/journal.pntd.0013611 (PMC12591400; doi:10.1371/journal.pntd.0013611)
Supplement: S1 Table — The RNA integrity number (RIN) is reported as an indicator of RNA quality, with higher values reflecting more intact RNA. RNA quality was assessed with Bioanalyzer 2100 Eukaryote total RNA Nano. (DOCX) [file pntd.0013611.s006.docx]

S1 Table. Pre-sequencing RNA quality control results provided by the sequencing facility (Novogene). The RNA integrity number (RIN) is reported as an indicator of RNA quality, with higher values reflecting more intact RNA. RNA quality was assessed with Bioanalyzer 2100 Eukaryote total RNA Nano.

| Customer Sample ID | Sample Type | Volume (µl) | Concentration (ng/µl) | Total Quantity (ng) | RIN/DIN |
| --- | --- | --- | --- | --- | --- |
| WA1 | RNA | 20 | 145.00 | 2900 | 6.9 |
| WA2 | RNA | 20 | 92.60 | 1852 | 7.2 |
| WA3 | RNA | 20 | 77.80 | 1556 | 7.0 |
| TX1 | RNA | 20 | 91.20 | 1824 | 4.0 |
| TX2 | RNA | 20 | 119.00 | 2380 | 8.9 |
| CO1 | RNA | 20 | 103.00 | 2060 | 7.2 |
| CO2 | RNA | 20 | 105.00 | 2100 | 7.3 |
| CO3 | RNA | 20 | 116.00 | 2320 | 7.1 |
| CA1 | RNA | 20 | 104.00 | 2080 | 5.6 |
| CA2 | RNA | 20 | 98.80 | 1976 | 5.3 |
| CA3 | RNA | 20 | 70.00 | 1400 | 6.3 |
| CA4 | RNA | 20 | 116.00 | 2320 | 6.1 |
| CA5 | RNA | 20 | 272.00 | 5440 | 8.8 |
| CA6 | RNA | 20 | 113.00 | 2260 | 4.9 |
| KWNR | RNA | 23 | 208.00 | 4784 | 9.4 |
