# Supplementary material for: Virome profiling of Culex tarsalis through small RNA-seq: A challenge of suboptimal samples
Source: PLoS Negl Trop Dis. 2025 Nov 3;19(11):e0013611. doi: 10.1371/journal.pntd.0013611 (PMC12591400; doi:10.1371/journal.pntd.0013611)
Supplement: S2 Table — The table reports the closest hits with their accession numbers. (DOCX) [file pntd.0013611.s007.docx]

S2 Table. BLAST analysis of Sanger sequencing results using blastn against the NCBI core nucleotide database. The table reports the closest hits with their accession numbers.

| Sample | Sequence length (bp) | Scientific Name | Max Score | Total Score | Query Cover | E value | Per. Ident | Acc. Len | Accession |
| --- | --- | --- | --- | --- | --- | --- | --- | --- | --- |
| MAR | 606 | Marma virus | 174 | 174 | 72% | 2.00E-38 | 68.85% | 3152 | MW434898.1 |
| CIV4 | 644 | Culex Iflavi-like virus 4 | 904 | 904 | 95% | 0.00% | 92.16% | 9723 | OM817535.1 |
| CN1 | 468 | Culex narnavirus 1 | 181 | 181 | 71% | 8.00E-41 | 70.96% | 3138 | MW434192.1 |
| WMV6 | 504 | Wuhan Mosquito Virus 6 | 247 | 247 | 82% | 3.00E-60 | 72.66% | 2441 | MW434433.1 |
| PCMV | 488 | Partitivirus-like Culex mosquito virus | 159 | 159 | 90% | 3.00E-34 | 67.65% | 1663 | MH188050.1 |
| CB2 | 1010 | Culex Bunyavirus 2 | 225 | 298 | 32% | 2.00E-53 | 77.59% | 7460 | MW434593.1 |
| HMV4* | 367 | Tombusviridae sp. | 107 | 172 | 74% | 1.00E-18 | 77.78% | 4967 | PP076491.1 |
|  |  | Hubei mosquito virus 4 | 104 | 175 | 74% | 1.00E-17 | 76.12% | 4952 | MW434930.1 |

*For HMV4, the two closest hits are included.
